# Phase I trial of the novel taxane BMS-184476 administered in combination with carboplatin every 21 days

**DOI:** 10.1038/sj.bjc.6601885

**Published:** 2004-06-22

**Authors:** J H Bilenker, J P Stevenson, M L Gallagher, D Vaughn, M B Cohen, P J O'Dwyer

**Affiliations:** 1 Abramson Family Cancer Center, University of Pennsylvania Cancer Center, 51 N. 39th St., MAB, Philadelphia, PA 19104, USA; 2 Bristol-Myers Squibb Pharmaceutical Research Institute, Princeton, NJ 08543, USA

**Keywords:** BMS-184476, carboplatin, taxane, hypersensitivity

## Abstract

The aim of the study was to determine the maximum-tolerated dose and dose-limiting toxicities for BMS-184476, in combination with carboplatin, in patients with advanced solid tumours and to describe any preliminary antitumour activity associated with this regimen. Patients received combination therapy with BMS-184476 given intravenously over 1 h followed by carboplatin administered over 30 min on day 1 of a 21-day cycle. In all, 28 patients received 146 cycles of BMS-184476 and carboplatin. Patients were enrolled at four dose levels: BMS-184476 (mg m^−2^)/carboplatin (mg min ml^−1^): 40/5, 50/5, 50/6 and 60/6. Dose-limiting toxicity at 60/6 was neutropenia. Among 27 evaluable patients, 11 demonstrated stable disease for a median of 8.5 cycles. In 22 patients, the pharmacokinetics of BMS-184476 appeared independent of dose of BMS-184476. The mean±s.e.m. values for clearance (Cl), volume of distribution at steady state and apparent terminal half-life of BMS-184476 in the four dose groups during cycle 1 were 192±25 ml min m^−2^, 377±69 l m^−2^ and 33.7±5.9 h, respectively. An increase in the dose of carboplatin from 5 to 6 mg min ml^−1^ may have decreased Cl of BMS-184476. BMS-184476 in combination with carboplatin was well tolerated at a dose of 50/6 and shows evidence of antitumour activity in a pretreated population.

BMS-184476 is a novel taxane, characterised by the substitution of a 7-methylthiomethyl ether group for the 7-hydroxyl group found on paclitaxel (Altstadt *et al*, 2001). BMS-184476 was developed to counter two common mechanisms of paclitaxel resistance: elimination through the P-glycoprotein efflux pump and resistance through tubulin mutations. BMS-184476 demonstrated greater activity than paclitaxel in a P-glycoprotein 170-mediated multidrug-resistant cell line as well as in the A2780/tax cell line (Rose *et al*, 2001). There has also been encouraging antitumour activity in human xenograft tumour models (Rose *et al*, 2001). The 7-methylthiomethyl ether substitution increases the solubility of the drug and reduces the amount of Cremophor EL® required as diluent. However, it is not clear as to whether these changes in formulation have resulted in less hypersensitivity and neurotoxicity than seen with commercially available taxanes.

Two phase I studies of BMS-184476 given as a single agent have been published. Hidalgo *et al* (2001) treated 34 patients at doses ranging from 20 to 80 mg m^−2^, every 21 days. Dose-limiting toxicities (DLTs) of neutropenia, febrile neutropenia, diarrhoea and mucositis established a maximum-tolerated dose (MTD) of 60 mg m^−2^. Plummer *et al* (2002) have reported results of a second phase I study in which BMS-184476 was given weekly for 3 consecutive weeks, every 28 days, although this schedule was later amended to a modified schedule of days 1 and 8 dosing every 21 days. In 53 treated patients, neutropenia emerged as the DLT and diarrhoea was a common nonhaematologic toxicity. These authors recommended a phase II dose of 50 mg m^−2^ on days 1 and 8 of 21-day cycle.

We conducted a phase I trial of BMS-184476 in combination with carboplatin on day 1 of a 21-day cycle. Combination regimens incorporating a taxane with a platinum salt are widely used in the treatment of solid tumours such as ovarian cancer, non-small-cell lung cancer (NSCLC) and other solid tumours. We investigated the pharmocokinetic profile of BMS-184476 in combination with carboplatin, and report preliminary evidence of antitumour activity of the combination. The goals of the study were to characterise the toxicities of the combination and to define the appropriate dose for phase II studies.

## MATERIALS AND METHODS

### Patient population

Adult patients were eligible for this study if they had a histologically confirmed advanced solid malignancy. Patients were required to have either measurable or evaluable disease, an ECOG performance status of ⩽2 and at least 4 intervening weeks since their last prior treatment (or 6 weeks for drugs with delayed toxicity). Enrolment was also conditional upon adequate end-organ function, defined by the corresponding laboratory values: an absolute granulocyte count ⩾1500 mm^−3^, platelet count ⩾100 000 mm^−3^, serum bilirubin ⩽2.0 mg dl^−1^; serum AST and ALT ⩽2.5 times institutional upper limits of normal (⩽5-fold the institutional upper limit of normal for patients with liver metastases) and serum creatinine ⩽1.5 times the institutional upper limit of normal (or measured or calculated creatinine clearance (Cl) ⩾60 ml min^−1^). All toxicity related to prior treatment was required to have recovered to baseline or ⩽Grade 1. The exclusion criteria included a history of severe hypersensitivity reactions (HSRs) to agents containing Cremophor EL® (polyoxyethylated castor oil), pre-existing neurologic toxicity greater than Grade 1 and active brain metastases.

This study was approved by the Institutional Review Board of the University of Pennsylvania and all enrolled patients provided written informed consent.

### Treatment plan

BMS-184476 was supplied in a 5 ml vial as a 15 mg ml^−1^ solution, in conjunction with a second vial containing aqueous Cremophor EL® solution as diluent. The solution was further diluted with 250 cm^3^ 5% dextrose or 0.9% sodium chloride. BMS-184476 was administered intravenously (i.v.) by infusion pump over 1 h on day 1 of 21-day cycle. Within an hour after completion of the BMS-184476 infusion, carboplatin was administered over 30 min.

The treatment protocol was amended to include routine hypersensitivity prophylaxis after reactions were noted in four of the first 17 subjects treated. This regimen consisted of diphenhydramine 50 mg, dexamethasone 10 mg and cimetidine 300 mg given i.v. 30 min prior to BMS-184476 administration.

Patients experiencing a DLT could remain on study upon recovery from toxicity to baseline or ⩽Grade 1. For patients with Grade 4 neutropenia lasting 5 or more days or febrile neutropenia, the BMS-184476 dose was decreased by one level (dose levels −1 and −2 were 30 and 25 mg m^−2^, respectively) with a concomitant dose reduction in carboplatin of 50%.

The carboplatin infusion was prepared from commercially available vials containing sterile aqueous solution, which were diluted with 5% dextrose injection or 0.9% sodium chloride injection. After antiemetic prophylaxis, which included a 5-HT3 inhibitor, carboplatin was given as a 30-min i.v. infusion on day 1 of the 21-day cycle. Antiemetic prophylaxis and treatment were employed, including a 5-HT3 antagonist, dexamethasone and metoclopramide.

Colony-stimulating factors were not administered prophylactically, but could be used at the discretion of the treating physician in the event of febrile neutropenia. Prophylactic and therapeutic use of erythropoietin was permitted.

Table 1

**Table 1:** Dose escalation schema

**Dose level**	**BMS-184476 (mg m^−2^)**	**Carboplatin AUC (mg min ml^−1^)**	**No. of patients**
1	40	5	7
2	50	5	6
3^a^	50	6	13
4	60	6	2

a Dose level expanded after MTD established at dose level 4. MTD=maximum-tolerated dose

summarises the dose escalation schedule administered, with the corresponding number of patients treated. Dose escalations were based on the occurrence of DLTs in the first cycle of treatment, defined as drug-related adverse events and graded according to the National Cancer Institute Common Toxicity Criteria 2.0. DLTs were defined as: absolute neutrophil count (ANC) <500 mm^−3^ for ⩾5 consecutive days or febrile neutropenia; platelet count <25 000 mm^−3^ or Grade 3/4 thrombocytopenia requiring transfusion; Grade 3/4 nausea and vomiting despite appropriate medical intervention; any other Grade 3/4 nonhaematologic toxicity except: fatigue/asthenia and transient arthralgia/myalgia.

If a DLT was observed in zero of three patients at a dose level, escalation to the next dose level was permitted. If a DLT was observed in one of the first three subjects at a dose level, three additional patients were treated at that level. If two or more subjects experienced a DLT at a dose level, that dose level was deemed the maximum dose level to be tested. If a DLT occurred, repeated cycles could be delayed for up to 14 days.

Patients with stable disease or response to therapy were continued on therapy. Responses were evaluated according to World Health Organization (WHO) criteria (World Health Organization, 1979).

### Patient monitoring

On the day of therapy, a medical history, physical examination, complete blood count (CBC), biochemical profile, electrocardiogram, urinalysis and chest X-ray were performed. Patients were subsequently monitored by twice weekly CBCs and weekly biochemical profiles. Patients with documented myelosuppression were followed by CBCs every 2 days until recovery. Physical examination as well as appropriate imaging procedures to assess the response of the patient's disease were performed and repeated every other course.

### Sample collection and analysis

Serial blood samples were collected predose and at 0.5, 1, 1.25, 1.5, 2, 2.5, 3, 4, 6, 24, 48 and 72 h after the start of the 1-h infusion of BMS-184476. Blood samples (5 ml) were collected from a peripheral vein, from a site contralateral to the BMS-184476 infusion. Within 1 h of collection, the plasma was separated by centrifugation at approximately 1000 r.p.m. for 15 min at 4°C. Plasma was stored at or below −20°C until analysis.

Plasma samples were analysed for concentrations of BMS-184476, its sulphoxide metabolites (BMS-246178 and BMS-246180), and paclitaxel by a validated HPLC method (Bristol-Myers Squibb Pharmaceutical Research Institute, Princeton, NJ, USA). The criteria for acceptance of an analytical run specified that the deviation of the predicted concentrations from the nominal concentrations for at least three-fourths of all calibration standards for an analyte and at least two-thirds of all quality control samples for an analyte be within ±15%. After the addition of internal standard, BMS-183061, to 1.0 ml of plasma, the sample was vortexed and loaded onto an unendcapped Cyano (CN-U) solid-phase extraction column (Varian, Harbor City, CA, USA). The compounds were eluted with 0.1% formic acid in methanol, the eluent was evaporated to dryness and the residue was reconstituted. Chromatographic separation of the compounds was achieved on a Zorbax RX-C18, 5 *μ*m, 4.6 × 250 mm^2^ column (Hewlett Packard Co., Wilmington, DE, USA) using a mobile phase consisting of 51.5% acetonitrile in water containing 10 mmol l^−1^ ammonium acetate (pH titrated to 5.0 with acetic acid) and 10 mmol l^−1^ tetramethlammonium hydroxide (pH titrated to 5.0 with acetic acid) at 40°C. The flow rate was 1.0 ml min^−1^. Detection was by ultraviolet absorbance at 227 nm. The lower limit of quantitation of the assay was 9.14 ng ml^−1^ for BMS-184476, 4.65 ng ml^−1^ for BMS-246178, 4.65 ng ml^−1^ for BMS-246180 and 8.54 ng ml^−1^ for paclitaxel.

### Pharmacokinetic and pharmacodynamic analyses

Estimates of pharmacokinetic parameters for BMS-184476 were derived from individual concentration–time data sets by noncompartmental analysis methods performed using the PKMENU application written in SAS version 6.12. (SAS, Cary, NC, USA) (Gibaldi and Perrier, 1982). The values of the maximum plasma concentration (*C*_max_) were recorded directly from experimental observations. The area under the plasma concentration *vs* time curve from time zero to the time of the last measurable concentration *T* (AUC_0–*T*_) was calculated using a combination of linear and log trapezoidal summations. The first-order rate constant of decline of BMS-184476 concentrations in the terminal phase of the plasma concentration–time data set, *λ*, was estimated by log-linear regression, using no weighting factor, of at least three data points yielding a minimum mean square error. The absolute value of *λ* was used to estimate the apparent terminal elimination half-life (*t*_1/2_). The last measurable concentration and the rate constant, *λ*, were used to extrapolate the AUC_0–*T*_ to estimate the area under the curve from time zero to infinity (AUC_0–*∞*_). The total body Cl was calculated by dividing the dose by AUC_0–*∞*_. The volume of distribution at steady state (*V*_SS_) was calculated using standard noncompartmental methods.

The relationships between BMS-184476 systemic exposure and toxicity as indicated by the percentage of decrease in the ANC were explored. The percentage of decrease in ANC was calculated as follows, for cycle 1:


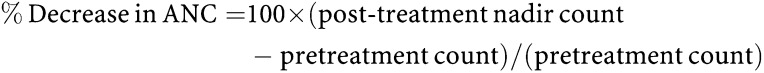


The relationship between BMS-184476 AUC_0–*∞*_ and the percentage of decrease in the ANC was evaluated using Kinetica 4.0.2 (InnaPhase Corporation, Philadelphia, PA, USA). Discrimination between pharmacodynamic models was guided by minimisation of the weighted sum of squares and standard errors for the pharmacodynamic parameters, examination of the dispersion of the residuals and use of the objective function, Akaike criteria and Schwartz criteria.

## RESULTS

### Patient characteristics

The demographic characteristics for patients entered on this study are shown in Table 2

**Table 2:** Patient characteristics

	***n***
Entered/evaluable	28/27
Male/female	15/13
Median age (range)	60 (33–78)
	
*Performance status*	
0	17
1	11
	
*Primary tumour site*	
Lung (NSCLC)	12
Colon	3
Gastric	3
Adenoidcystic	2
Melanoma	2
Mesothelioma	2
Biliary tract	1
Ovary	1
Pancreas	1
Peritoneal	1
Sarcoma	1
	
*Prior treatment*	
Previous chemotherapy	11
Previous radiation	1
Previous chemotherapy and radiation	4
No Previous chemotherapy or radiation	12

. In all, 28 patients with a median age of 60 years were enrolled (range 33–78 years). Patients varied with respect to previous therapy and 11 had no prior chemotherapy or radiation. A total of 146 cycles of BMS-184476 and carboplatin were given, for a median of two per patient (range 1–25).

### Dose-limiting toxicity

Patients were enrolled at four combination dose levels, summarised in Table 1. At the first dose level, one of six patients experienced Grade 4 neutropenia in the first cycle that lasted ⩾5 days, representing a DLT. This patient had melanoma and was previously treated with dacarbazine, cisplatin, carmustine, and tamoxifen (so-called ‘Dartmouth regimen’). Dose escalation continued when no additional DLTs occurred at this dose level. No DLTs were observed in the second and third dose levels, initially. At the fourth dose level, both patients treated demonstrated DLTs of neutropenia lasting ⩾5 days. A 74-year-old patient man with NSCLC and no prior therapies experienced Grade 4 neutropenia in cycle 1 and went off study. A 76-year-old female with NSCLC and no prior therapies also experienced Grade 4 neutropenia in cycle 1. This patient remained on study for an additional eight cycles, despite Grade 3 or 4 neutropenia in each, after appropriate delay and dose modification, without complication. These DLTs halted the dose-escalation portion of the study. Subsequent expansion of the third dose level resulted in two additional DLTs, described below.

### Haematologic toxicity

Haematologic toxicity was seen commonly in patients at all dose levels, in the first cycle of treatment and in subsequent cycles. Table 3a

**Table 3:** (a) Haematologic toxicity during cycle 1 and (b) worst haematologic toxicity (all cycles)

		**Neutropenia**					**Thrombocytopenia**				
**Dose level**	**No. of patients**	**0**	**1**	**2**	**3**	**4**	**0**	**1**	**2**	**3**	**4**
*(a) Haematologic toxicity*											
1	7	4	—	1	1	1	6	—	—	1	—
2	6	—	3	—	3	—	5	1	—	—	—
3	13	1	3	2	3	4^a^	12	1	—	—	—
4	2	—	—	—	—	2	—	—	—	—	—
*(b) Worst haematologic toxicity*											
1	40	23	4	9	3	1	27	11	—	1	—
2	16	—	4	1	7	4	8	7	1	—	—
3	75	11	5	11	24	24	51	20	2	2	—
4	15	3	—	—	4	8	5	8	2	—	—

a Lasted <5 days in two patients. Two DLTs seen after dose expansion.

summarises results from cycle 1. At the second dose level, Grade 3 neutropenia was seen in three of six patients. No episodes of Grade 4 neutropenia were seen in the first six patients at the third dose level.

The first two patients entered on the fourth dose level experienced dose-limiting neutropenia, as described above, halting the escalation phase of the study. Upon expanding the third dose level, four of seven patients demonstrated Grade 4 neutropenia, although two of these episodes lasted less than 5 days and were not considered DLTs. Two patients experienced neutropenia, meeting the criteria for DLT. The first of these was a 52-year-old man with mesothelioma and one prior course of chemotherapy who suffered no febrile or infectious complications. The second was a 56-year-old female with NSCLC and no prior treatments who went on to receive seven additional cycles of therapy at modified doses.

As illustrated in Table 3b, there appeared to be some cumulative haematologic toxicity. Grade 4 neutropenia was seen at all dose levels and reported in a total of 37 of 149 cycles administered; 24 of these episodes occurred in the 13 patients treated at the third dose level.

### Nonhaematologic toxicity

There were no instances of Grade 3 or 4 nonhaematologic toxicity in the first cycle. Fatigue, nausea, vomiting and diarrhoea were common, although of manageable severity (Table 4

**Table 4:** Nonhaematologic toxicity (cycle 1)

		**Fatigue**				**Nausea**				**Vomiting**				**Diarrhoea**			
**Dose level**	**No. of patients**	**0**	**1**	**2**	**3**	**0**	**1**	**2**	**3**	**0**	**1**	**2**	**3**	**0**	**1**	**2**	**3**
1	7	4	1	2	—	2	3	2	—	3	4	—	—	5	2	—	—
2	6	3	—	3	—	3	2	1	—	3	2	1	—	5	—	1	—
3	13	7	4	2	—	6	6	1	—	12	—	1	—	10	3	—	—
4	2	1	1	—	—	—	—	—	—	—	—	—	—	—	—	—	—

). When subsequent cycles were considered, one episode of Grade 4 vomiting was reported in a patient with advanced pancreatic cancer. An episode of Grade 4 gastrointestinal bleeding, which was deemed to be disease related, was reported in a patient with advanced colon cancer. Sensory neuropathy was reported by four patients and limited to Grade 1 during 16 cycles. Motor neuropathy was reported by two patients and described as Grade 1 in three cycles and Grade 2 in two cycles. No trend of cumulative neurotoxicity was apparent in these patients. Eight patients reported alopecia that was limited to Grade 2. Grade 1 arthralgias and myalgias were reported in 10 and two cycles, respectively.

One patient at dose level 1 experienced a Grade 2 HSR in his second cycle of treatment. A second patient at dose level 2 developed a Grade 2 HSRs in the first cycle of treatment. A third patient treated at dose level 2 developed Grade 2 hypersensitivity during the first two cycles of treatments. A fourth patient treated at dose level 3 demonstrated a Grade 1 HSR during cycle 1 and a Grade 2 HSR during cycle 2. No additional episodes were seen after the implementation of a prophylactic regimen consisting of diphenhydramine 50 mg, dexamethasone 10 mg and cimetidine 300 mg given i.v. 30 min prior to the BMS-184476 infusion.

### Patient benefit

A total of 11 patients demonstrated stable disease as their best response. They included patients with NSCLC (6), colon (1), gall bladder (1), gastric (1) and ovarian (1) cancers and melanoma (1). Patients with stable disease received a median of 8.5 cycles (range: 4–25). A patient with NSCLC and no previous therapy was treated at the first dose level and received 25 cycles of therapy over 18 months. Two other patients with NSCLC, both without prior therapy, exhibited stable disease for 14 cycles each. None of the six NSCLC patients exhibiting stable disease had received prior therapy.

### Pharmacokinetics and pharmacodynamics

Evaluable plasma concentration–time profiles were obtained from 22 patients during the first cycle of treatment; one patient at the 60/6 dose level had no samples collected and one patient at the 40/5 dose level, one patient at the 50/5 dose level and four patients at the 50/6 dose level were excluded from pharmacokinetic evaluation because an insufficient number of samples were collected to permit a reliable analysis. Plasma concentrations were quantifiable in all patients through 48–72 h (Table 5

**Table 5:** Worst selected nonhaematologic toxicities (per course)

	**No. of courses (%)**				
	**0**	**1**	**2**	**3**	**4**
Fatigue	86	30	29	1	—
Nausea	95	42	8	1	—
Alopecia	110	19	17	—	—
Constipation	121	10	15	—	—
Diarrhoea	127	14	5	—	—
Vomiting	128	14	4	—	1
Sensory neuropathy	130	16	—	—	—
Peripheral oedema	133	13	—	—	—
Arthralgia	136	10	—	—	—
Anorexia	138	6	1	1	—
Hypersensitivity	139	2	5	—	—
Motor neuropathy	141	3	2	—	—
GI bleeding	143	2	—	—	1
Myalgia	144	2	—	—	—
Stomatitis	144	2	—	—	—
Rash	145	1	—	—	—
Tinnitus	145	1	—	—	—
Nystagmus	145	1	—	—	—
Hand–foot	145	1	—	—	—

).

**Figure 1:**
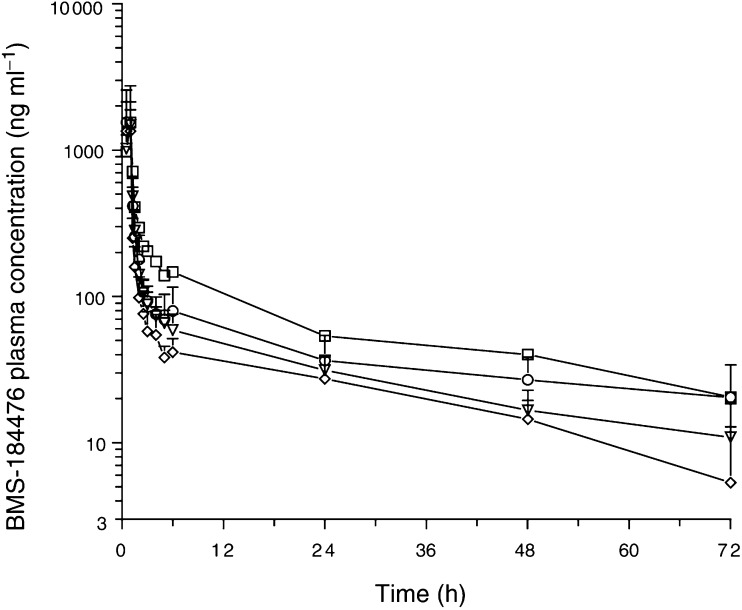
Mean concentration–time profiles of BMS-184476 after the administration of BMS-184476 (mg m^−2^)/carboplatin (mg min ml^−1^) at dose levels of 40/5 (◊), 50/5 (▿), 50/6 (○), 60/6 (□). Error bars indicate standard deviation.

The mean (s.d.) plasma concentration–time profiles of BMS-184476 are shown in Figure 1

and mean BMS-184476 pharmacokinetic parameters are listed in Table 6

**Table 6:** Pharmacokinetic parameters of BMS-184476 in patients during cycle 1

**Dose group^b^**	**No. of patients**	***C*_max_ (ng ml^−1^)**	**AUC_0*-∞*_ (h ng ml^−1^)**	**Cl (ml min m^−2^)**	***t*_1/2_ (h)**	***V*_SS_ (l m^−2^)**
40/5	6	1566±874	3413±920	212±73	33.6±15.6	388±231
50/5	5	1415±414	3776±429	218±22	28.9±11.9	361±152
50/6	9	1964±1255	5507±2791	181±73	43.2±18.6	476±266
60/6	2	1545^c^	6613^c^	155^c^	28.8^c^	284^c^

a Values are the mean±s.d.

b BMS-184476 (mg m^−2^)/carboplatin (mg min ml^−1^).

c s.d. not presented because *n*=2.

. The pharmacokinetics of BMS-184476 appeared independent of the dose of BMS-184476. The mean±s.e.m. values for Cl, volume of distribution at steady state and apparent terminal half-life of the four dose groups during cycle 1 were 192±25 ml min m^−2^, 377±69 l m^−2^ and 33.7±5.9 h, respectively. Cl of BMS-184476 was compared at carboplatin doses of 5 and 6 mg min ml^−1^, with mean±s.d. values of 215±54 (*n*=11) and 177±67 ml min m^−2^ (*n*=11), respectively. This difference did not achieve statistical significance (*P*=0.15). At the recommended phase II dose of 50/6, interpatient variability in the principal pharmacokinetic parameters was moderate, with coefficients of variation percentages of 40, 56 and 43 for CL, *V*_SS_ and *t*_1/2_, respectively, and there was a 4.2-fold range of AUC_0–*∞*_ values. The relationship of *C*_max_ or AUC_0–*∞*_ to a dose of BMS-184476 could not be evaluated because of the limited range of doses administered with either 5 or 6 mg min ml^−1^ of carboplatin. The metabolites BMS-246178, BMS-246180 and paclitaxel were sporadically quantifiable with concentrations much less than BMS-184476.

**Figure 2:**
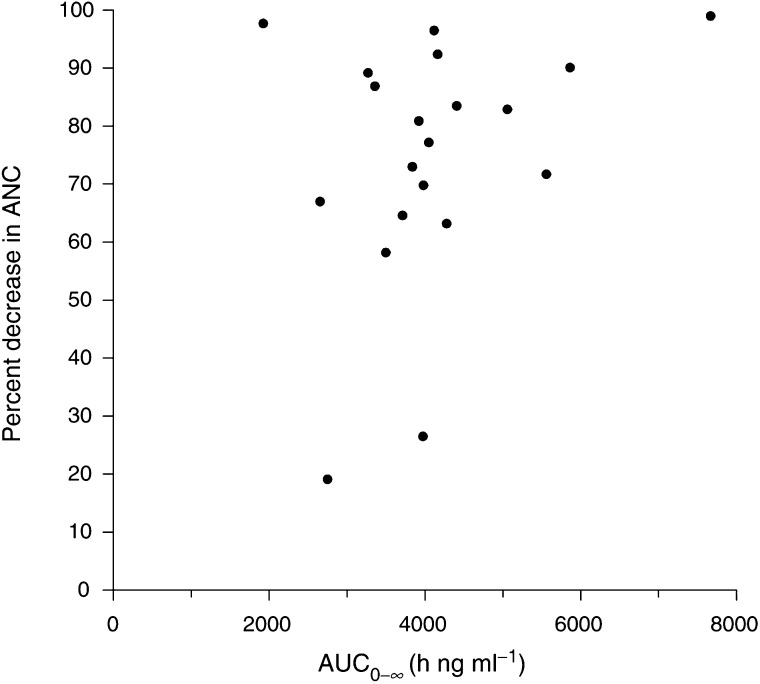
Scatter plot showing the relationship between BMS-184476 AUC_0–*∞*_ and the percentage of decrease in ANC during course 1 (•).

A scatter plot of the pharmacodynamic relationship between the percentage of decrease in ANC during course 1 and the BMS-184476 exposure as determined by the AUC_0–*∞*_ is shown in Figure 2

. The relationship in this study was not well described by models of drug action, including linear, log linear, *E*_max_ or sigmoid *E*_max_.

## DISCUSSION

Dose-limiting toxicity of neutropenia was seen at the highest dose level planned for this study, at doses of BMS-184476 60 mg m^−2^ and carboplatin AUC of 6 mg min ml^−1^. An expansion of the penultimate dose level – BMS-184476 50 mg m^−2^ and carboplatin AUC of 6 mg min ml^−1^ – revealed two additional DLTs and two other episodes of Grade 4 neutropenia, not meeting the criteria for DLT in cycle 1. These Grade 4 adverse events did not occur in heavily pretreated patients. Moreover, some cumulative myelotoxicity was observed in the 13 patients treated at the expanded dose level (Table 3b). For these reasons, we recommend that further study of this combination in the phase II setting should begin at dose of BMS-184476 50 mg m^−2^ and carboplatin AUC of 6 mg min ml^−1^, with the recognition that intraindividual dose modification may be required as the number of cycles increase.

The non-haematologic toxicities we observed for this combination were similar to those previously reported for BMS-184476 given alone and in combination. Febrile neutropenia, diarrhoea and/or mucositis were dose limiting at doses of 70 and 80 mg m^−2^ given every 3 weeks in the single-agent study reported by Hidalgo *et al* (2001). One of 15 patients treated at 60 mg m^−2^ (deemed the MTD) exhibited Grade 4 febrile neutropenia and Grade 3 diarrhoea. A second phase I study of an alternative dosing schedule, in which BMS-184476 was given on days 1 and 8 of 21-day cycle, also revealed unacceptable neutropenia at doses exceeding 50 mg m^−2^ (Plummer *et al*, 2002). A combination study of BMS-184476 and doxorubicin reported three DLTs of neutropenia at doses of doxorubicin 50 mg m^−2^ and BMS-184476 at 40 mg m^−2^ given every 3 weeks, prompting the investigation of other dosing schedules (Sessa *et al*, 2001). Our group recently reported results of a phase I study of BMS-184476 given in combination with cisplatin given every 3 weeks. Grade 4 neutropenia was seen in three of 18 patients in cycle 1, at doses of BMS-184476 60 mg m^−2^ and cisplatin 75 mg m^−2^. In the BMs-184476/cisplatin study, diarrhoea, nausea and vomiting also met the criteria for DLTs. The toxicities were substantially less in the carboplatin combination and full doses of both BMS-184476 and carboplatin could be administered to most patients. Thus, the combination of BMS-184476 and carboplatin caused a similar degree of neutropenia at comparable BMS-184476 doses tested in single-agent studies (Hidalgo *et al*, 2001; Plummer *et al*, 2002). The addition of carboplatin did not appear to increase this toxicity profile. Moreover, the incidence and severity of thrombocytopenia observed at all dose levels was low, suggesting that BMS-184476 may provide the same platelet sparing effects as taxol, when combined with carboplatin (Belani *et al*, 1999).

At the initiation of this study, a premedication regimen for the prevention of HSR was not given, in light of reports from single-agent trials and the expectation that lower Cremophor EL content would markedly decrease the incidence of such reactions. However, four patients developed ⩽Grade 2 hypersensitivity reactions in our study, prompting the administration of a prophylactic premedication regimen. A regimen consisting of i.v. diphenhydramine 50 mg, dexamethasone 10 mg and cimetidine 300 mg prevented additional HSRs in the patients with previous episodes and in previously untreated patients. Although HSRs may be less frequent and less severe than with paclitaxel, we recommend that prophylactic premedication for all patients in future studies of BMS-184476.

The pharmacokinetics of BMS-184476 combined with 5 mg min ml^−1^ of carboplatin in this study were similar to those of BMS-184476 given as a single agent and were characterised by a large volume of distribution and a long apparent terminal elimination half-life (Plummer *et al*, 2002). Although there is some indication that the increase of dose of carboplatin from 5 to 6 mg min ml^−1^ may have resulted in a decreased CL of BMS-184476, the difference is not statistically significant. Also, there was no indication that carboplatin affected the metabolism of BMS-184476 to its sulphoxide metabolites (BMS-246178 and BMS-246180) or paclitaxel. The pharmacokinetics of BMS-184476 in combination with carboplatin in this study appeared linear as was observed for BMS-18476 as a single agent (Plummer *et al*, 2002). This is in contrast to the nonlinear pharmacokinetic behaviour of paclitaxel (Gibaldi and Perrier, 1982; Henningsson *et al*, 2001).

In this study of BMS-184476 combined with carboplatin, the relationship during course 1 between haematologic toxicity as determined by the percentage of decrease in ANC and the BMS-184476 exposure as determined by the AUC_0–*∞*_ was not well described by several models of drug action. In a previous study of BMS-184476 as a single agent, the relationship was well described by a sigmoid *E*_max_ model (Rose *et al*, 2001). The lack of fit to a sigmoid *E*_max_ model might be explained by the narrower dose range in this study (40–60 mg m^−2^) as compared to the previous study (20–80 mg m^−2^) that resulted in a narrower range of exposure. However, the relationship might then be expected to be described by a linear or log-linear model. Another possible explanation is that the AUC_0–*∞*_ of BMS-184476 alone is not adequate to describe the decrease in ANC when BMS-184476 is combined with carboplatin.

Patient benefit was limited to stable disease in 11 patients. Six of these had NSCLC that had not been previously treated. When achieved, stable disease appeared fairly durable, with patients receiving a median of 8.5 cycles (range: 4–25). Six of the patients carried diagnoses of NSCLC, all previously untreated. Three NSCLC patients also remained on therapy for the greatest number of cycles, for 25, 14 and 14 cycles, respectively.

In summary, we demonstrated that a combination regimen of BMS-184476 at 50 mg m^−2^ and carboplatin AUC of 6 mg min ml^−1^ given on day 1 of a 21-day cycle had a manageable toxicity profile, with neutropenia representing the most frequent adverse event. Durable stable disease in six patients with NSCLC justify further study of this regimen in this population.
